# Preoperative hypoalbuminemia was associated with acute kidney injury in high-risk patients following non-cardiac surgery: a retrospective cohort study

**DOI:** 10.1186/s12871-019-0842-3

**Published:** 2019-09-02

**Authors:** Nan Li, Hong Qiao, Jing-Fei Guo, Hong-Yun Yang, Xue-Ying Li, Shuang-Ling Li, Dong-Xin Wang, Li Yang

**Affiliations:** 1 Department of Critical Care Medicine, Peking University First Hospital, No.8 Xishiku Street, Xicheng District, Beijing, 100034 China; 2 Critical Care Nephrology Research Center, Peking University First Hospital, No.8 Xishiku Street, Xicheng District, Beijing, 100034 China; 3 Department of Anesthesiology, Fuwai Hospital, Chinese Academy of Medical Sciences, No.167 North Lishi Road, Xicheng District, Beijing, 100037 China; 4 Clinical Laboratory, Peking University First Hospital, No.8 Xishiku Street, Xicheng District, Beijing, 100034 China; 5 Department of Biostatistics, Peking University First Hospital, No.8 Xishiku Street, Xicheng District, Beijing, 100034 China; 6 Department of Nephrology, Peking University First Hospital, No.8 Xishiku Street, Xicheng District, Beijing, 100034 China

**Keywords:** Hypoalbuminemia, Acute kidney injury, Non-cardiac surgery, Prognosis

## Abstract

**Background:**

Acute kidney injury (AKI) is a common complication following non-cardiac surgery with adverse short- and long- term morbidity and mortality. Evidence shows that hypoalbuminemia is associated with increased AKI risk in patients with infectious diseases and cancer and following cardiac surgery and transplant surgery. However, little evidence is available on non-cardiac surgery population. Thus, we investigated the association between preoperative hypoalbuminemia and AKI following non-cardiac surgery.

**Methods:**

We retrospectively assessed perioperative risk factors and preoperative serum albumin concentration in 729 consecutive adult patients who underwent non-cardiac surgery from July 1, 2017, to June 30, 2018. Each patient was categorized according to maximal Kidney Disease Improving Global Outcomes criteria based on creatinine changes and urine output within the first week after surgery. Multivariate Logistic regression models were used to analyze the association between preoperative hypoalbuminemia and postoperative AKI.

**Results:**

Of 729 patients, 188 (25.8%) developed AKI. AKI incidence was higher in patients with preoperative serum albumin < 37.5 g/L than in those with preoperative serum albumin ≥37.5 g/L [35.9% (98/273) vs. 19.7% (90/456), *P* < 0.001]. Multivariate logistic regression analysis showed that preoperative serum albumin < 37.5 g/L (odds ratio 1.892; 95% confidence interval 1.238–2.891; *P* = 0.003) was independently associated with postoperative AKI. Patients with preoperative serum albumin < 37.5 g/L tended to have a higher but not significant ratio in AKI stage 2 (2.6% vs 1.1%, *P* = 0.144) and much higher ratio in AKI stage 3 (4.8% vs 0.7%, *P* < 0.001) than those with preoperative serum albumin ≥37.5 g/L. AKI patients had a higher in-hospital mortality rate [6.9% (13/188) vs. 0.2% (1/541), *P* < 0.001]. Kaplan-Meier analysis revealed that the cumulative survival rate decreased with increasing AKI severity (*P* < 0.001). Postoperative AKI was also associated with other worse outcomes, such as prolonged mechanical ventilation [53.4 (33.0, 73.8) vs 14.7 (11.1, 18.3) hours, P < 0.001], intensive care unit stay [4.0 (3.1, 4.9) vs 2.0 (1.8, 2.3) days, P < 0.001], postoperative hospital stay [17.8 (14.8, 20.9) vs 12.3 (11.3, 13.3) days, P < 0.001], and higher total cost [13,453 (8538, 20,228) vs 11,306 (6277, 16,400) dollars, P < 0.001].

**Conclusions:**

Preoperative hypoalbuminemia was independently associated with AKI after non-cardiac surgery, and postoperative AKI was associated with poor outcomes.

**Electronic supplementary material:**

The online version of this article (10.1186/s12871-019-0842-3) contains supplementary material, which is available to authorized users.

## Background

Acute kidney injury (AKI) is a common complication after non-cardiac surgery with an incidence ranging from 6.8 to 39.3% according to different patient populations [1, 2]. Several underlying susceptibilities, procedures, or exposures have been identified to be risk factors of postoperative AKI occurrence, such as older age, chronic kidney disease, diabetes, sepsis, major surgery, and hemodynamic instability [3]. Recent evidence demonstrated that AKI was independently associated with longer length of hospital stay and higher rate of 30-day hospital readmission, 1-year end-stage renal disease, and mortality with more severe stage of AKI relating to poorer outcomes after non-cardiac surgery [4, 5]. Unfortunately, an effective treatment for AKI in the intensive care unit has not been established [6], strongly suggesting that early recognition of and adjusting for risk factors would be beneficial for high-risk patients.

Hypoalbuminemia is a well-established risk factor for increased morbidity and mortality in acutely ill patients [7]. The association between hypoalbuminemia and AKI is consistently evident in many observational studies conducted across different clinical settings, mainly focusing on infectious diseases, cancer, cardiac surgery, and transplant surgery [8–12]. Although the underlying mechanisms for this association are not fully elucidated, serum albumin may play a protection role in the maintenance of renal perfusion, preservation of proximal tubular integrity and function, binding of endogenous toxins and nephrotoxic drugs, prevention of oxidative damage, and delivery of protective lysophosphatidic acid [13, 14]. Therefore, we assumed that preoperative hypoalbuminemia might be associated with an increased risk of AKI following non-cardiac surgery. However, limited data are currently available on this topic [15–18]. Thus, this study aimed to investigate the association between preoperative serum albumin concentration and AKI occurrence in high-risk patients following non-cardiac surgery.

## Methods

### Ethics and consent

Ethical approval (2018–137) was provided by the Clinical Research Ethics Committee of Peking University First Hospital on July 4, 2018. Because of the retrospective nature of the study and no patient follow-up was performed, the ethics committee agreed to waive written informed consent. This study was performed in accordance with Strengthening the Reporting of Observational Studies in Epidemiology (STROBE) criteria [see Additional file 1: Table S1].

### Patients

The study period was from July 1, 2017, to June 30, 2018, with the following inclusion criteria: (1) adult patients (age ≥ 18 years), (2) undergoing non-cardiac surgery, (3) admitted to the surgical intensive care unit (SICU), and (4) at a high risk of postoperative AKI. High-risk patients referred to as patients having at least one of the following conditions: (1) preoperative comorbidities, including hypertension, diabetes mellitus, coronary heart disease, congestive heart failure, cerebrovascular disease, chronic kidney disease, lung disease, or liver disease; (2) major surgery, defined as surgery duration ≥2 h; (3) ongoing organ dysfunction, defined as the sequential organ failure assessment (SOFA) score ≥ 2 from one single organ system. Patients with any of the following criteria were excluded: (1) chronic kidney disease stage 5 or requiring long-term dialysis; (2) surgery involving kidney, such as nephrectomy, partial nephrectomy, nephroureterectomy, or kidney transplantation; (3) AKI events before surgery; and (4) incomplete clinical data. Our medical center is a teaching hospital affiliated with a university, which provides tertiary care and has about 1600 beds.

### Definitions of outcomes

The primary end point was postoperative AKI development. Postoperative AKI and its severity was defined according to Kidney Disease Improving Global Outcomes criteria using the maximal change in serum creatinine compared with the preoperative baseline values and urine output during the first 7 postoperative days [19].

The secondary end points were the postoperative use of mechanical ventilation (MV) and its duration, length of ICU and postoperative hospital stay, number of postoperative complications other than AKI, total cost, and in-hospital mortality.

Other main postoperative complications were pulmonary infection, pleural effusion, pulmonary atelectasis, respiratory failure, surgical bleeding, new-onset arrhythmia, acute myocardial infarction, congestive heart failure, hemodynamic insufficiency, stroke, acute liver injury, disseminated intravascular coagulation, ileus, anastomotic leakage, intra-abdominal abscess, wound infection, wound dehiscence, urinary tract infection, sepsis, digestive tract bleeding, and venous thromboembolism [see Additional file 2: Table S2].

### Other data collection

Patients’ data were searched through the electronic medical records system of our hospital. Perioperative data were collected including demographic characteristics (age, sex), body mass index (BMI), medical history, American Society of Anesthesiology (ASA) physical status classification, as well as preoperative nephrotoxin exposure. Other data collected were preoperative clinical laboratory data, such as hemoglobin, albumin, baseline serum creatinine, and B-type natriuretic peptide (BNP). Intraoperative data included type and duration of surgery, emergency surgery, duration of anesthesia, maximal lactate, minimal hemoglobin, estimated blood loss, use of vasopressors, volume of artificial colloids infusion, and fluid balance. Postoperative data before AKI included new onset of nephrotoxin exposure, sepsis, use of vasopressors, minimal hemoglobin, maximal lactate and BNP, perioperative blood transfusion, and non-renal SOFA score within 24 h of ICU admission.

### Statistical analysis

Preoperative serum albumin concentration were firstly compared between patients with and without AKI by independent samples t-test. Then to determine its cutoff value for postoperative AKI occurrence, receiver operating characteristic curve analysis was performed. The patients were divided into two groups according to the occurrence of AKI or hypoalbuminemia. Quantitative variables with normal distribution were compared by independent samples t-test; numeric data with abnormal distribution were compared by Mann-Whitney U test. Qualitative variables were compared by chi-squared test or Fisher’s exact test. Time-to-event data were analyzed with Kaplan-Meier survival analysis, with difference between groups compared by log- rank test. After testing for collinearity, perioperative variables with *P <* 0.10 and number of events > 10 in the univariate analyses for AKI occurrence were included in multivariate logistic regression model (backward stepwise method) to identify independent risk factors for AKI. Furthermore, baseline variables unbalanced between patients with or without hypoalbuminemia were entered into propensity score matching. Then, patients were matched 1:1 based on their scores using nearest-neighbor matching with the tolerance being 0.02. Thereafter, logistic regression analysis was performed to find out the association between hypoalbuminemia and AKI. Two-sided *P* values < 0.05 were regarded as statistically significant. The SPSS v21.0 software package was used for statistical processing. (SPSS Inc., Chicago, IL, USA).

## Results

**Fig. 1:**
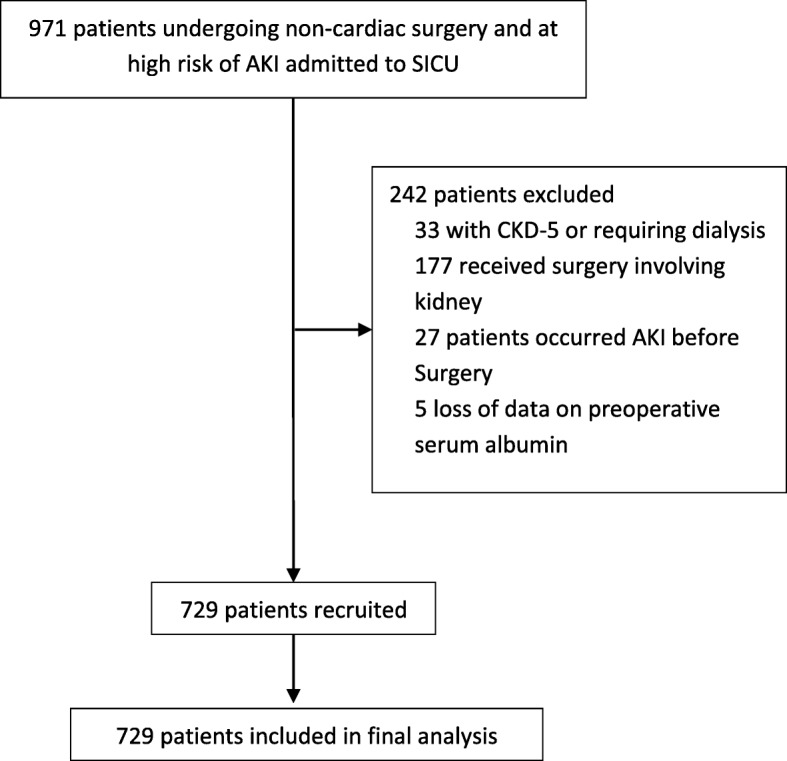
Flow diagram of the study

During the study period, a total of 971 patients at a high risk of AKI undergoing non-cardiac surgery were admitted to SICU; among them, 729 met the inclusion/exclusion criteria and were included in the final statistical analysis (Fig. 1). Baseline and perioperative data were listed in Tables 1 and 2. Among the enrolled patients, 188 (25.8%) developed postoperative AKI stages 1, 2, and 3, which accounted for 21.9, 1.6, and 2.2%, respectively. Patients with postoperative AKI had significantly lower level of preoperative albumin compared with patients without AKI (36.7 ± 6.3 vs 39.3 ± 6.0 g/L, *P* < 0.001). The cutoff value of preoperative serum albumin for postoperative AKI occurrence was 37.5 g/L determined by the Youden index (P < 0.001, area under the curve [AUC] = 0.624) with a sensitivity of 0.54, specificity of 0.67, and positive predictive value of 0.36 [see Additional file 3: Figure S1].

**Table 1:** Preoperative variables

	Total (*n* = 729)	Without postoperative AKI (*n* = 541)	With postoperative AKI (*n* = 188)	P value	Preoperative albumin ≥37.5 g/L^a^ (*n* = 456)	Preoperative albumin < 37.5 g/L^a^ (*n* = 273)	*P* value
Age (y)	67 ± 16	66 ± 16	71 ± 14	< 0.001	66 ± 15	69 ± 14	0.056
Male sex	432 (59.3%)	318 (58.8%)	114 (60.6%)	0.655	276 (60.5%)	156 (57.1%)	0.368
BMI (kg/m^2^)	24.1 ± 4.5	24.0 ± 4.6	24.6 ± 4.4	0.139	24.7 ± 4.3	23.2 ± 4.7	< 0.001
Preoperative comorbidities							
Diabetes mellitus	194 (26.6%)	136 (25.1%)	58 (30.9%)	0.127	123 (27.0%)	71 (26.0%)	0.775
Hypertension	399 (54.7%)	287 (53.0%)	112 (59.6%)	0.122	260 (57.0%)	139 (50.9%)	0.109
Coronary heart disease	195 (26.7%)	138 (25.5%)	57 (30.3%)	0.199	130 (28.5%)	65 (23.8%)	0.165
Congestive heart failure	23 (3.2%)	14 (2.6%)	9 (4.8%)	0.133	13 (2.9%)	10 (3.7%)	0.544
Cerebrovascular disease	140 (19.2%)	99 (18.3%)	41 (21.8%)	0.293	94 (20.6%)	46 (16.8%)	0.212
Chronic kidney disease	23 (3.2%)	9 (1.7%)	14 (7.4%)	< 0.001	8 (1.8%)	15 (5.5%)	0.005
Lung disease ^b^	68 (9.3%)	51 (9.4%)	17 (9.0%)	0.876	41 (9.0%)	27 (9.9%)	0.686
Liver disease ^c^	20 (2.7%)	16 (3.0%)	4 (2.1%)	0.548	10 (2.2%)	10 (3.7%)	0.240
Malignant neoplasm	474 (65.0%)	349 (64.5%)	125 (66.5%)	0.624	247 (65.1%)	177 (64.8%)	0.935
Peripheral vascular disease	37 (5.1%)	24 (4.4%)	13 (6.9%)	0.182	21 (4.6%)	16 (5.9%)	0.455
ASA classification				< 0.001			< 0.001
I	12 (1.6%)	12 (2.2%)	0 (0.0%)		7 (1.5%)	5 (1.8%)	
II	275 (37.7%)	224 (41.4%)	51 (27.1%)		202 (44.3%)	73 (26.7%)	
III	404 (55.4%)	284 (52.5%)	120 (63.8%)		238 (52.2%)	166 (60.8%)	
IV	38 (5.2%)	21 (3.9%)	17 (9.0%)		9 (2.0%)	29 (10.6%)	
Preoperative Hb (g/L) ^d^	124 ± 23	125 ± 22	119 ± 26	0.007	133 ± 18	108 ± 23	< 0.001
Preoperative albumin (g/L) ^d^	38.6 ± 6.2	39.3 ± 6.0	36.7 ± 6.3	< 0.001	42.5 ± 3.0	32.1 ± 4.2	< 0.001
Preoperative albumin < 37.5 g/L	273 (37.4%)	175 (32.3%)	98 (52.1%)	< 0.001	__	__	__
Baseline serum creatinine (umol/L) ^e^	80.4 ± 26.3	78.2 ± 22.2	86.7 ± 35.1	0.002	81.5 ± 23.6	78.5 ± 30.4	0.163
Preoperative BNP (pg/ml) ^f^	98 (43, 190)	84 (38, 166)	126 (61, 220)	0.001	66 (31, 133)	140 (72, 257)	< 0.001
Radiocontrast exposure ^g^	76 (10.4%)	48 (8.9%)	28 (14.9%)	0.020	44 (9.6%)	32 (11.7%)	0.375
On ACEI/ARB	62 (8.5%)	48 (8.9%)	14 (7.4%)	0.546	38 (8.3%)	24 (8.8%)	0.830
Smoking habit ^h^	136 (18.7%)	108 (20.0%)	28 (14.9%)	0.124	94 (20.6%)	42 (15.4%)	0.079

Data are presented as mean ± SD, median (interquartile range), or number of patients (percentage) and compared by independent samples t-test, Mann-Whitney U test or chi-squared test/Fisher’s exact test respectively

*ACEI* angiotensin converting enzyme inhibitor, *ARB* angiotensin receptor blocker, *ASA* American Society of Anesthesiologists, *BMI* body mass index, *BNP* B-type natriuretic peptide, *cTNI* cardio-troponin, *Hb* hemoglobin

^a^The cutoff value of preoperative albumin for postoperative AKI was determined by the Youden index of the ROC curve [see Additional file 3: Figure S1]

^b^Including chronic obstructive pulmonary disease, asthma, and pulmonary fibrosis

^c^Including any kind of chronic hepatitis and liver cirrhosis

^d^Measured within 3 days before surgery

^e^Determined by the minimal value of serum creatinine measured within 3 months before admission and in hospital before surgery; if neither value was available, the modification of diet in renal disease formula was adopted to estimate the baseline serum creatinine according to the Kidney Disease Improving Global Outcomes guideline

^f^Measured in 366 patients before surgery

^g^Including patients who had radiocontrast exposure within 7 days before surgery

^h^Smoking for more than 10 cigarettes per day for more than 1 year, including current or past smokers

**Table 2:** Intra- and postoperative variables

	Total (*n* = 729)	Without postoperative AKI (*n* = 541)	With postoperative AKI (*n* = 188)	*P* value	Preoperative albumin ≥37.5 g/L^a^ (*n* = 456)	Preoperative albumin < 37.5 g/L^a^ (*n* = 273)	*P* value
Duration of anesthesia (min)	258 (174, 338)	253 (169, 337)	277 (192, 347)	0.053	243 (167, 330)	272 (201, 364)	0.002
Duration of surgery (min)	171 (97, 249)	165 (92, 245)	192 (110, 256)	0.062	158 (88, 242)	189 (121, 272)	0.002
Emergency surgery	64 (8.8%)	37 (6.8%)	27 (14.4%)	0.002	17 (3.7%)	47 (17.2%)	< 0.001
Open surgery ^b^	288 (53.9%)	193 (50.0%)	95 (64.2%)	0.003	147 (45.1%)	141 (67.8%)	< 0.001
Type of surgery							
General surgery ^c^	330 (45.3%)	229 (42.3%)	101 (53.7%)	0.007	180 (39.5%)	150 (54.9%)	< 0.001
Neurosurgery	18 (2.5%)	15 (2.8%)	3 (1.6%)	0.585	10 (2.2%)	8 (2.9%)	0.535
Thoracic surgery	52 (7.1%)	38 (7.0%)	14 (7.4%)	0.846	33 (7.2%)	19 (7.0%)	0.888
Urologic surgery	195 (26.7%)	162 (29.9%)	33 (17.6%)	0.001	160 (35.1%)	35 (12.8%)	< 0.001
Gynecological surgery	25 (3.4%)	17 (3.1%)	8 (4.3%)	0.470	6 (1.3%)	19 (7.0%)	< 0.001
Orthopedic surgery	52 (7.1%)	38 (7.0%)	14 (7.4%)	0.846	29 (6.4%)	23 (8.4%)	0.294
Vascular surgery	15 (2.1%)	10 (1.8%)	5 (2.7%)	0.552	11 (2.4%)	4 (1.5%)	0.383
ENT surgery	8 (1.1%)	6 (1.1%)	2 (1.1%)	> 0.999	3 (0.7%)	5 (1.8%)	0.158
Others ^d^	24 (3.3%)	18 (3.3%)	6 (3.2%)	0.928	18 (3.9%)	6 (2.2%)	0.200
Intraoperative maximal lactate (mmol/L) ^e^	1.1 (0.8, 1.5)	1.1 (0.8, 1.5)	1.1 (0.8, 1.6)	0.995	1.1 (0.8, 1.5)	1.1 (0.8, 1.5)	0.421
Intraoperative minimal Hb (g/L) ^e^	112 ± 26	113 ± 26	111 ± 27	0.568	118 ± 25	104 ± 25	< 0.001
Intraoperative management							
Use of vasopressors ^f^	175 (24.1%)	116 (21.6%)	59 (31.4%)	0.007	116 (21.6%)	59 (31.4%)	0.007
Volume of artificial colloid infusion (ml)	500 (500, 1000)	500 (500, 1000)	500 (500, 1000)	0.858	500 (500, 1000)	500 (500, 1000)	0.618
Estimated blood loss (ml)	100 (10, 300)	100 (10, 300)	100 (10, 300)	0.138	100 (10, 300)	100 (10, 400)	0.034
Positive fluid balance (ml)	2202 ± 1605	2201 ± 1579	2204 ± 1679	0.982	2093 ± 1516	2382 ± 1731	0.023
Postoperative variables before AKI ^g^							
Nephrotoxin exposure							
Glycopeptides	46 (6.3%)	35 (6.5%)	11 (5.9%)	0.764	17 (3.7%)	29 (10.6%)	< 0.001
Aminoglycoside	0 (0.0%)	0 (0.0%)	0 (0.0%)	–	0 (0.0%)	0 (0.0%)	–
NSAIDs	387 (53.1%)	292 (54.0%)	95 (50.5%)	0.415	265 (58.1%)	122 (44.7%)	< 0.001
Hemolysis	1 (0.1%)	1 (0.2%)	0 (0.0%)	> 0.999	0 (0.0%)	1 (0.4%)	0.374
Rhabdomyolysis	1 (0.1%)	1 (0.2%)	0 (0.0%)	> 0.999	0 (0.0%)	1 (0.4%)	0.374
Sepsis	47 (6.4%)	29 (5.4%)	18 (9.6%)	0.043	12 (2.6%)	35 (12.8%)	< 0.001
Use of vasopressors ^f^	77 (10.6%)	53 (9.8%)	24 (12.8%)	0.254	41 (9.0%)	36 (13.2%)	0.074
Minimal Hb (g/L)	103 ± 25	105 ± 26	99 ± 21	0.008	109 ± 27	94 ± 18	< 0.001
Maximal BNP (pg/ml)	247 (130, 456)	244 (128, 443)	264 (142, 513)	0.265	209 (115, 373)	322 (188, 564)	< 0.001
Maximal lactate (mmol/L)	1.9 (1.3, 2.7)	1.9 (1.3, 2.7)	1.8 (1.3, 2.8)	0.751	1.9 (1.4, 2.7)	1.8 (1.3, 2.7)	0.377
Perioperative blood transfusion ^*h*^	186 (25.5%)	131 (24.2%)	55 (29.3%)	0.172	89 (19.5%)	97 (35.5%)	< 0.001
Non-renal SOFA within 24 h ICU admission	2 (1, 3)	2 (1, 3)	2 (2, 4)	0.005	2 (1, 3)	3 (1, 4)	0.001
Postoperative AKI	188 (25.8%)	–	–	–	90 (19.7%)	98 (35.9%)	< 0.001

Data are presented as mean ± SD, median (interquartile range), or number of patients (percentage) and compared by independent samples t-test, Mann-Whitney U test or chi-squared test/Fisher’s exact test respectively

*BNP* B-type natriuretic peptide, *ENT* ear, nose and throat, *Hb* hemoglobin, *NSAIDs* non-steroidal anti-inflammatory drugs, *SOFA* sequential organ failure assessment score

^a^ The cutoff value of preoperative albumin for postoperative AKI was determined by the Youden index of the ROC curve [see Additional file 3: Figure S1]

^b^ Open or laparoscopic surgery referred to 534 patients

^c^ Abdominal surgery, such as gastrointestinal, hepatobiliary, and pancreatic surgery

^d^ Thyroid or breast surgery

^e^ Measured by arterial blood gas analysis

^f^ Including use of phenylephrine, norepinephrine, epinephrine, and dopamine

^g^ Occurred before start of AKI

^*h*^ Perioperative blood product transfusion, including packed red blood cell, plasma, and platelet

The incidence of AKI in patients with serum albumin < 37.5 g/L (35.9% [98/273]) was significantly higher than those with serum albumin ≥37.5 g/L (19.7% [90/456]) (*P* < 0.001). Univariate analysis showed that preoperative serum albumin < 37.5 g/L was strongly associated with the occurrence of postoperative AKI (OR 2.277; 95% CI 1.624–3.194; P *<* 0.001). In the multivariate logistic regression model (backward), preoperative serum albumin < 37.5 g/L was identified to be independently associated with postoperative AKI (OR 1.892; 95% CI 1.238–2.891; P *<* 0.001). Other independent risk factors for AKI included age (OR 1.018; 95% CI 1.004–1.033; *P =* 0.013), radiocontrast exposure (OR 1.843; 95% CI 1.031–3.293; *P =* 0.039), baseline creatinine (OR 1.016; 95% CI 1.008–1.025; *P <* 0.001), ASA classification (OR 1.719; 95% CI 1.193–2.477; *P =* 0.004), and intraoperative use of vasopressors (OR 1.680; 95% CI 1.065–2.648; *P =* 0.026) (Table 3). After matching for age, BMI, history of chronic kidney disease, preoperative hemoglobin, and ASA classification, 161 pairs of patients with or without hypoalbuminemia were well balanced in their baseline variables except for malignant neoplasm and ASA classification [see Additional file 4: Table S3; Additional file 5: Table S4]. Logistic regression analysis once again revealed that preoperative serum albumin < 37.5 g/L was independently associated with postoperative AKI (OR 3.085; 95% CI 1.649–5.771; *P <* 0.001) [see Additional file 6: Table S5].

**Table 3:** Independent risk factors for postoperative AKI

	Univariate Logistic model ^a^		Multivariate Logistic model ^b^	
	OR (95% CI)	*P* value	OR (95% CI)	*P* value
Age (y)	1.022 (1.010–1.034)	< 0.001	1.018 (1.004–1.033)	0.013
History of chronic kidney disease	4.756 (2.023–11.180)	< 0.001	–	–
Sepsis	1.869 (1.013–3.451)	0.046	–	–
Radiocontrast exposure ^c^	1.797 (1.091–2.961)	0.021	1.843 (1.031–3.293)	0.039
Baseline creatinine (umol/L) ^d^	1.011 (1.005–1.017)	< 0.001	1.016 (1.008–1.025)	< 0.001
Preoperative Hb (g/L) ^e^	0.990 (0.983–0.997)	0.004	–	–
Preoperative albumin < 37.5 g/L ^e^	2.277 (1.624–3.194)	< 0.001	1.892 (1.238–2.891)	0.003
ASA classification	1.966 (1.472–2.625)	< 0.001	1.719 (1.193–2.477)	0.004
Open surgery	1.792 (1.213–2.650)	0.003	–	–
Emergency surgery	2.284 (1.349–3.869)	0.002		
General surgery ^f^	1.582 (1.133–2.208)	0.007	–	–
Duration of surgery (every 1 h increase)	1.047 (0.976–1.124)	0.202	–	–
Intraoperative use of vasopressors ^g^	1.660 (1.146–2.404)	0.007	1.680 (1.065–2.648)	0.026
Non-renal SOFA within 24 h ICU admission	1.133 (1.044–1.230)	0.003	–	–

*ASA* American Society of Anesthesiologists, *CI* confidence interval, *Hb* hemoglobin, *OR* odds ratio, *SOFA* sequential organ failure assessment score

^a^ Perioperative variables with *P <* 0.10 in the univariate analyses by independent samples t test, Mann-Whitney U test, chi-squared test, or Fisher’s exact test were included, except preoperative BNP, duration of anesthesia, and postoperative minimal Hb before AKI because of collinearity

^b^ Backward: LR

^c^ Including patients who had radiocontrast exposure within 7 days before surgery

^d^ Determined by the minimal value of serum creatinine measured within 3 months before admission and in hospital before surgery; if neither value was available, the modification of diet in renal disease formula was adopted to estimate the baseline serum creatinine according to the Kidney Disease Improving Global Outcomes guideline

^e^ Measured within 3 days before surgery

^f^ Abdominal surgery, such as gastrointestinal, hepatobiliary, and pancreatic surgery

^g^ Including use of phenylephrine, norepinephrine, epinephrine, and dopamine

Moreover, for severity of AKI, patients with preoperative serum albumin < 37.5 g/L tended to have a higher but not significant ratio in AKI stage 2 (2.6% vs 1.1%, *P* = 0.144) and a much higher ratio in AKI stage 3 (4.8% vs 0.7%, *P* < 0.001) than those with preoperative serum albumin ≥37.5 g/L.

To determine the cause of hypoalbuminemia, we further analyzed preoperative nutritional status using criteria of nutritional risk screening 2002 (NRS 2002). The results showed that patients with preoperative serum albumin < 37.5 g/L had significantly increased NRS score [4 (2, 4) vs 1 (1, 2), *P* < 0.001] and had a much higher ratio of NRS score ≥ 3 (77.5% vs 15.5%, *P* < 0.001).

**Fig. 2:**
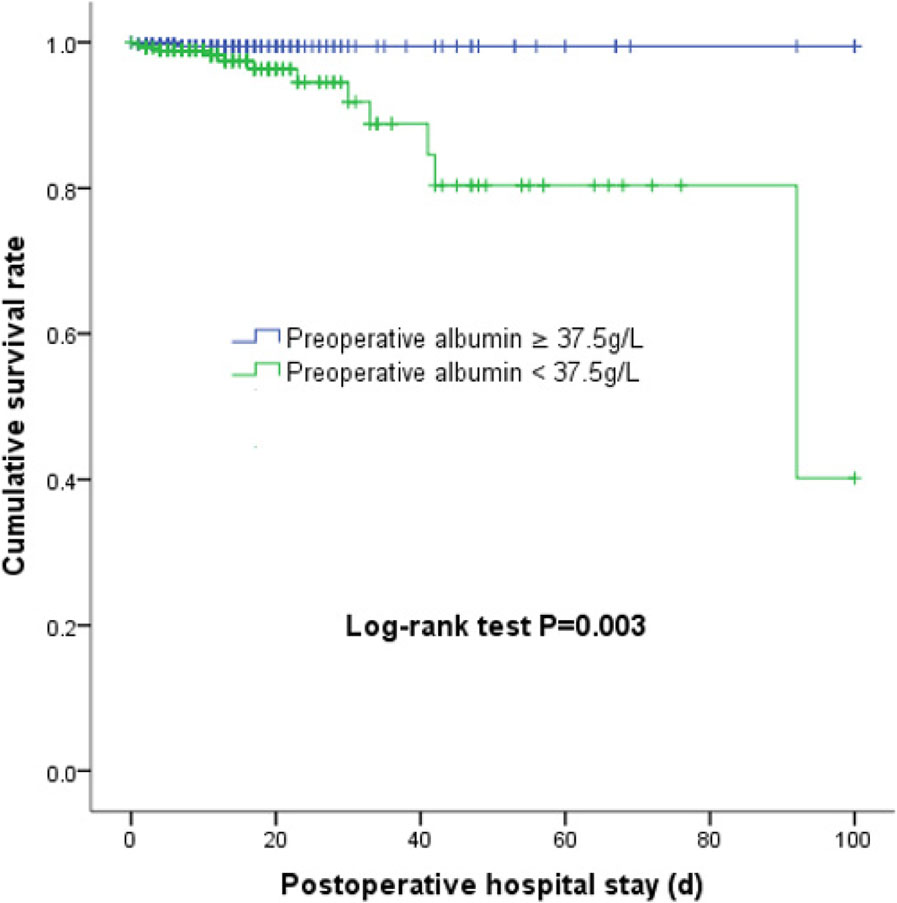
Cumulative survival rate of patients with preoperative serum albumin < 37.5 or ≥ 37.5 g/L.

**Fig. 3:**
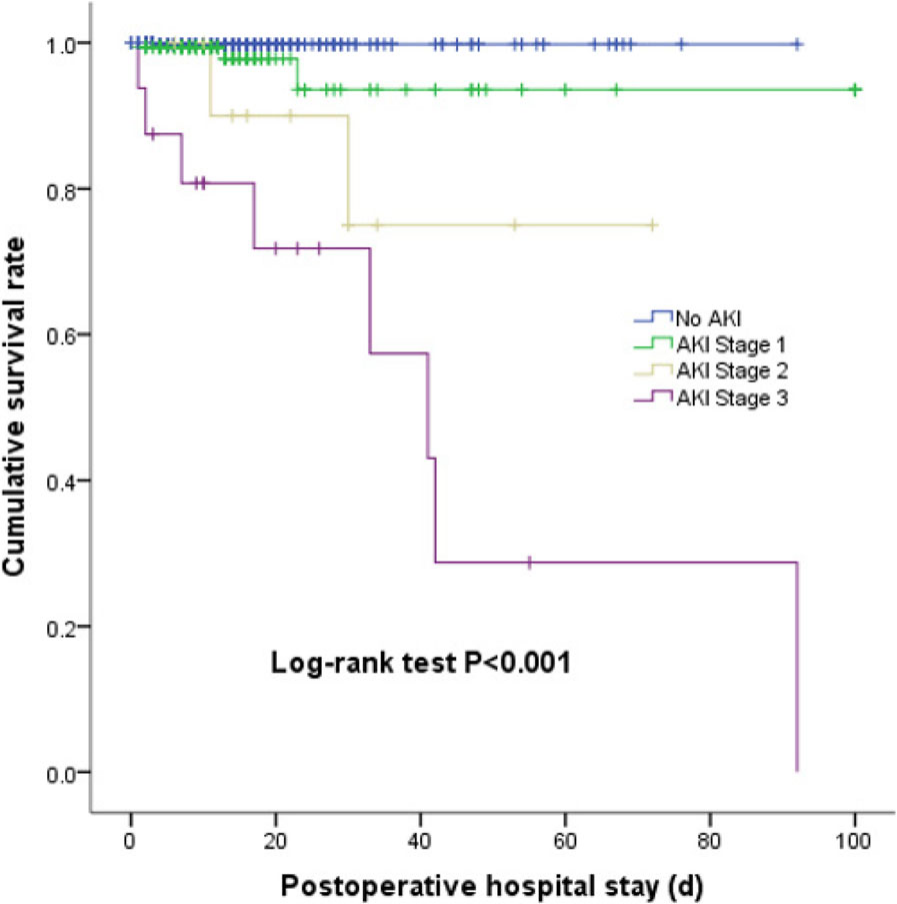
Cumulative survival rate of patients with stages 1–, 2, and 3 AKI during postoperative hospital stay

Of all included patients, 14 patients (1.9%) died during hospital stay. Patients with preoperative serum albumin < 37.5 g/L had a mortality rate of 4.4%, which was much higher than 0.4% in patients with preoperative serum albumin ≥37.5 g/L (*P* < 0.001). The cumulative survival rate was also lower in patients with hypoalbuminemia (*P* = 0.003) (Fig. 2). Compared with that in non-AKI patients, the mortality rate was significantly higher in AKI patients (6.9% [13/188] vs. 0.2% [1/541]; P < 0.001). Kaplan-Meier analysis revealed that the cumulative survival rate decreased with increasing AKI severity (*P* < 0.001) (Fig. 3). In addition, postoperative AKI was associated with other worse outcomes, such as prolonged mechanical ventilation [53.4 (33.0, 73.8) vs 14.7 (11.1, 18.3) hours, *P* < 0.001], higher rate of other postoperative complications [0 (0, 2) vs 0 (0, 0), *P* < 0.001], ICU stay [4.0 (3.1, 4.9) vs 2.0 (1.8, 2.3) days, *P* < 0.001], postoperative hospital stay [17.8 (14.8, 20.9) vs 12.3 (11.3, 13.3) days, *P* < 0.001], and higher total cost [13,453 (8538, 20,228) vs 11,306 (6277, 16,400) dollars, *P* < 0.001] (Table 4, Additional file 2: Table S2). We also further analyzed AKI patients, preoperative hypoalbuminemia (< 37.5 g/L) was associated with more use of MV (72.4% [71/98] vs. 56.7% [51/90]; *P* = 0.024), longer ICU stay [4.5 (3.3, 5.7) vs 3.4 (2.1, 4.8) days, *P* = 0.027], higher occurrence of postoperative complications [1 (0, 3) vs 0 (0, 1), P < 0.001], and higher mortality (11.2% [11/98] vs. 2.2% [2/90]; *P* = 0.020) and total cost [15,160 (10,345, 22,221) vs 12,111 (6262, 17,763) dollars, *P* = 0.011] (Table 4). All the dataset of our study are available [see Additional file 7: Dataset].

**Table 4:** Postoperative outcomes

	Total (*n* = 729)	All patients (n = 729)		P value	AKI patients (*n* = 188)		P value
		Without postoperative AKI (*n* = 541)	With postoperative AKI (*n* = 188)		Preoperative albumin ≥37.5 g/L (*n* = 90)	Preoperative albumin < 37.5 g/L (*n* = 98)	
On MV in ICU	390 (53.6%)	268 (49.6%)	122 (64.9%)	< 0.001	51 (56.7%)	71 (72.4%)	0.024
Duration of MV (h) ^*a*^	26.9 (19.8, 34.0)	14.7 (11.1, 18.3)	53.4 (33.0, 73.8)	< 0.001	55.4 (21.5, 89.4)	51.9 (26.5, 77.4)	0.835
Length of ICU stay (d) ^*b*^	2.5 (2.3, 2.8)	2.0 (1.8, 2.3)	4.0 (3.1, 4.9)	< 0.001	3.4 (2.1, 4.8)	4.5 (3.3, 5.7)	0.027
Number of postoperative complications except AKI ^*c*^	0 (0, 1)	0 (0, 0)	0 (0, 2)	< 0.001	0 (0, 1)	1 (0, 3)	< 0.001
Postoperative hospital stay (d) ^*d*^	13.7 (12.6, 14.8)	12.3 (11.3, 13.3)	17.8 (14.8, 20.9)	< 0.001	16.9 (11.8, 22.0)	18.7 (15.2, 22.2)	0.158
In-hospital mortality	14 (1.9%)	1 (0.2%)	13 (6.9%)	< 0.001	2 (2.2%)	11 (11.2%)	0.020
Total cost (10,000 dollars)	1.2 (0.7, 1.7)	1.1 (0.6, 1.6)	1.3 (0.9, 2.0)	< 0.001	1.2 (0.6, 1.8)	1.5 (1.0, 2.2)	0.011

Data are presented as number of patients (percentage), or median (interquartile range), unless otherwise indicated

*ICU* intensive care unit, *MV* mechanical ventilation

^*a*^ Results of patients requiring postoperative mechanical ventilation. Data were analyzed by Kaplan-Meier analysis and compared by log-rank test; results are presented as average (95% confidence interval);

^*b*^ Result of patients admitted to ICU. Data were analyzed by Kaplan-Meier analysis and compared by log-rank test; results are presented as average (95% confidence interval)

^*c*^ Including pulmonary infection, pleural effusion, atelectasis, respiratory failure, acute myocardial infarction, congestive heart failure, new-onset arrhythmia, hemodynamic insufficiency, stroke, venous thromboembolism, ileus, intra-abdominal abscess, wound infection, urinary tract infection, sepsis, surgical bleeding, digestive tract bleeding, acute liver injury, disseminated intravascular coagulation, anastomotic leakage, and wound dehiscence [see Additional file 2: Table S2]

^*d*^ Data were analyzed by Kaplan-Meier analysis and compared by log-rank test; results are presented as average (95% confidence interval)

## Discussion

Results of this retrospective study showed that preoperative hypoalbuminemia was independently associated with AKI occurrence in high-risk patients following non-cardiac surgery. In addition, more severe AKI stage was found in hypoalbuminemic patients. In accordance with previous reports of outcomes after non-cardiac surgery [1, 2, 20], the in-hospital mortality rate in AKI patients (6.9%) was very much higher than that in patients without AKI (0.2%). Other outcomes, such as ICU, postoperative hospital stay, and total cost, were also much worse in AKI patients. Furthermore, AKI patients with hypoalbuminemia had even more detrimental outcomes.

Cumulative evidence have shown that hypoalbuminemia is an important risk factor for postoperative AKI in various clinical settings [8–12]. However, in surgical settings, studies were mainly focused on cardiac surgery and transplant surgery [11, 12, 21–23]. Few studies have examined the effect of preoperative hypoalbuminemia on postoperative AKI patients undergoing non-cardiac surgery. Kim et al. [15] conducted a retrospective study enrolling 4718 patients who underwent partial or total gastrectomy for gastric cancer, and they revealed that patients with preoperative hypoalbuminemia, defined as < 40 g/L, had a significantly increased risk for AKI (OR 1.4; 95% CI 1.11–1.77). In patients following hip fracture surgery or total knee arthroplasty, after adjustment for confounders, early postoperative hypoalbuminemia has been shown to be strongly associated with AKI with a cutoff value of < 29 g/L and < 30 g/L [16, 17]. Recently, Kim et al. found that a preoperative serum albumin level < 38 g/L was independently associated with AKI (OR 2.465; CI 1.310–4.640) and mortality (OR 3.223; CI 1.959–5.305) in patients undergoing brain tumor surgery [18]. The finding from our study that preoperative hypoalbuminemia had a significant relationship with postoperative AKI was consistent with the results above. The cutoff value for hypoalbuminemia in our patients was 37.5 g/L, well above the usually accepted definition for hypoalbuminemia. Thus, our results suggested that with even a little decrease in preoperative serum albumin concentration, a higher incidence of postoperative AKI would occur in high-risk patients undergoing non-cardiac surgery.

Several possible mechanisms underlie this association. As a scavenger of radical oxygen species, combined with its anti-inflammation effects, albumin limits tubular cell apoptosis [24, 25]. Recent data have suggested that the integrity of the glycocalyx might be compromised in patients with hypoalbuminemia leading to loss of oncotic pressure gradients and barrier function, fluid leakage into the interstitium, and microvascular flow alterations [26, 27]. Moreover, ligation of endogenous toxin, modulation of nitric oxide and pharmacokinetic and pharmacodynamic effects of albumin also play an important role in renal protection [28, 29].

Albumin cutoff values vary between studies, and we attributed this difference to various study populations and types of surgery. In our study, as mentioned above, the cutoff value of 37.5 g/L had a sensitivity of 0.54, specificity of 0.67, and positive predictive value of 0.36; in patients undergoing brain tumor surgery, the cutoff value of 38 g/L had a similar sensitivity of 0.54, but lower specificity of 0.27 and positive predictive value of 0.04, which might be partly explained by the low incidence of AKI (1.8%) [18]. However, the AUC appeared to be similar with 0.624 in our non-cardiac surgery patients, 0.653 in hip fracture patients [16], and 0.684 in brain tumor patients [18]. Considering the possible negative association between serum albumin and AKI occurrence as reflected by research in patients undergoing cardiac surgery [11], we assumed that patients with a higher risk of postoperative AKI, such as having several comorbidities or undergoing general surgery [4], might have a lower tolerance threshold of serum albumin for AKI occurrence, thus requiring higher levels of serum albumin to protect perioperative renal function.

Currently, increasing amount of data revealed that postoperative AKI occurrence is associated with short-term adverse outcomes such as higher mortality and longer ICU and hospital stay [1, 2, 4, 11], which was also confirmed by our study. Furthermore, in AKI patients, preoperative hypoalbuminemia was associated with more use of MV, longer ICU stay, higher occurrence of postoperative complications, and higher mortality and total cost.

Unfortunately, there is still no effective treatment for AKI at present. Therefore, early recognition of high-risk patients and prevention of postoperative AKI become the first priority in clinical practice. Basic and clinical studies mentioned above indicated a potential benefit of correcting hypoalbuminemia for renal protection. Excitingly, Lee et al. [30] had made a step further. They recently performed a randomized controlled trial evaluating the effects of exogenous 20% human albumin solution vs saline on the incidence of postoperative AKI in adult patients with hypoalbuminemia (< 40 g/L) undergoing off-pump coronary artery bypass surgery. Their results have demonstrated that the incidence of postoperative AKI was lower in the intervention group than in the control group (17.6% vs 31.7%; *P* = 0.031). Multivariate logistic regression analysis revealed a renal-protective effect of albumin infusion with nearly 60% risk of AKI decreased (OR = 0.42, 95% CI: 0.21–0.83; *P* = 0.012). However, further studies are needed to address the results in the future, especially in patients undergoing non-cardiac surgery. Another way to increase preoperative serum albumin level is optimization of nutritional status. As shown in our study, 77.5% of hypoalbuminemic patients had preoperative NRS score ≥ 3, which indicated that malnutrition might be an important contributor to the occurrence of hypoalbuminemia. Until now, several studies have demonstrated significantly better results in overall and infectious complications in patients undergoing preoperative nutritional therapy [31–33]. However, data on the association between nutritional support and postoperative AKI were limited. Therefore, more work is needed to verify the effects of optimizing nutritional status on AKI, especially for patients undergoing non-cardiac surgery.

This study has major limitations. First, although we considered many perioperative AKI-related variables in our analysis, the effects of non-investigated factors could not be totally excluded. Second, given the lack of statistical power, subgroup analyses for the association of different preoperative albumin levels with AKI were not performed. Finally, in view of the retrospective and observational nature of this study, a causal relationship between preoperative hypoalbuminemia and risk of postoperative AKI could not be determined.

## Conclusions

Our results showed that preoperative hypoalbuminemia was independently associated with AKI in high-risk patients following non-cardiac surgery, and postoperative AKI was associated with adverse prognosis. Prospective trials are needed to further identify the association between hypoalbuminemia and AKI and explore the potential beneficial effects of albumin infusion or specific nutritional therapy on postoperative AKI prevention.

## Additional files

Additional file 1: **Table S1.** STROBE Statement. Checklist of our cohort study, which demonstrates STROBE Statement Checklist of our cohort study. (DOCX 28 kb)

Additional file 2: **Table S2.** Occurrence of other postoperative complications. Demonstrates the occurrence and definitions of other postoperative complications. (DOCX 21 kb)

Additional file 3: **Figure S1.** Preoperative serum albumin receiver operating characteristic curve for discriminating critically ill subjects with or without AKI. Demonstrates preoperative serum albumin receiver operating characteristic curve for discriminating patients with or without AKI. (DOCX 29 kb)

Additional file 4: **Table S3.** Preoperative variables after propensity score matching. Description: Demonstrates preoperative variables between patients with or without hypoalbuminemia after propensity score matching. (DOCX 20 kb)

Additional file 5: **Table S4.** Intra- and postoperative variables after propensity score-matching. Demonstrates intra- and postoperative variables between patients with or without hypoalbuminemia after propensity score-matching. (DOCX 21 kb)

Additional file 6: **Table S5.** Independent risk factors for postoperative AKI after propensity score matching. Demonstrates the independent risk factors for postoperative AKI after propensity score matching. (DOCX 16 kb)

Additional file 7: Dataset. Relevant data underlying the main results. (XLSX 413 kb)

## Data Availability

All data generated or analyzed during this study are included in this published article and its supplementary information files.

## Abbreviations

AKI: Acute kidney injury
ASA: American Society of Anesthesiology
AUC: Area under the curve
BMI: Body mass index
BNP: B-type natriuretic peptide
MV: Mechanical ventilation
NRS: Nutritional risk screening
SICU: Surgical intensive care unit
SOFA: Sequential organ failure assessment
