# Supplementary material for: Preoperative hypoalbuminemia was associated with acute kidney injury in high-risk patients following non-cardiac surgery: a retrospective cohort study
Source: BMC Anesthesiol. 2019 Sep 2;19:171. doi: 10.1186/s12871-019-0842-3 (PMC6719349; doi:10.1186/s12871-019-0842-3)
Supplement: Supplementary file 2 — Table S2. Occurrence of other postoperative complications. Demonstrates the occurrence and definitions of other postoperative complications. (DOCX 21 kb) [file 12871_2019_842_MOESM2_ESM.docx]

**Table S2. Occurrence of other postoperative complications.**

| Complications | Total (n=729) | Without postoperative AKI (n=541) | With postoperative AKI (n=188) | P value |
| --- | --- | --- | --- | --- |
| Pulmonary complications |  | | | |
| Pulmonary infection *^a^* | 57 (7.8%) | 20 (3.7%) | 37 (19.7%) | <0.001 |
| Pleural effusion *^b^* | 18 (2.5%) | 6 (1.1%) | 12 (6.4%) | <0.001 |
| Atelectasis *^c^* | 11 (1.5%) | 2 (0.4%) | 9 (4.8%) | <0.001 |
| Respiratory failure *^d^* | 38 (5.2%) | 15 (2.8%) | 23 (12.2%) | <0.001 |
| Surgical bleeding *^e^* | 9 (1.2%) | 5 (0.9%) | 4 (2.1%) | 0.247 |
| New onset arrhythmia *^f^* | 32 (4.4%) | 16 (3.0%) | 16 (8.5%) | 0.001 |
| Acute myocardial infarction *^g^* | 12 (1.6%) | 7 (1.3%) | 5 (2.7%) | 0.200 |
| Congestive heart failure *^h^* | 16 (2.2%) | 4 (0.7%) | 12 (6.4%) | <0.001 |
| Hemodynamic insufficiency *^i^* | 71 (9.7%) | 36 (6.7%) | 35 (18.6%) | <0.001 |
| Stroke *^j^* | 9 (1.2%) | 3 (0.6%) | 6 (3.2%) | 0.011 |
| Ileus *^k^* | 7 (1.0%) | 3 (0.6%) | 4 (2.1%) | 0.077 |
| Anastomotic leakage *^l^* | 19 (2.6%) | 11 (2.0%) | 8 (4.3%) | 0.113 |
| Intra-abdominal abscess *^m^* | 13 (1.8%) | 3 (0.6%) | 10 (5.3%) | <0.001 |
| Acute liver injury *^n^* | 32 (4.4%) | 16 (3.0%) | 16 (8.5%) | 0.001 |
| Wound infection *^o^* | 11 (1.5%) | 4 (0.7%) | 7 (3.7%) | 0.007 |
| Wound dehiscence *^p^* | 4 (0.5%) | 2 (0.4%) | 2 (1.1%) | 0.275 |
| Urinary tract infection *^q^* | 11 (1.5%) | 7 (1.3%) | 4 (2.1%) | 0.487 |
| Sepsis *^r^* | 40 (5.5%) | 15 (2.8%) | 25 (13.3%) | <0.001 |
| Disseminated intravascular coagulation *^s^* | 15 (2.1%) | 7 (1.3%) | 8 (4.3%) | 0.031 |
| Digestive tract bleeding *^t^* | 9 (1.2%) | 4 (0.7%) | 5 (2.7%) | 0.054 |
| Venous thromboembolism |  | | | |
| Pulmonary embolism *^u^* | 1 (0.1%) | 1 (0.2%) | 0 (0.0%) | >0.999 |
| Deep venous thrombosis *^v^* | 30 (4.1%) | 17 (3.1%) | 13 (6.9%) | 0.025 |

Data are presented as number of patients (percentage).

*^a^* Presence of at least one of the following manifestations (increased or color-changed sputum, new or changed pulmonary infiltrates, fever, leukocyte count > 12,000/mm^3^) and required antibiotic therapy;

*^b^* Confirmed by chest X-ray or ultrasound examination and required therapeutic intervention (drainage, aspiration, and/or diuresis after albumin administration);

*^c^* Confirmed by chest X-ray examination, with or without oxygen desaturation, and required therapeutic intervention (oxygenation inhalation, physical therapy, and/or mechanical ventilation);

*^d^* Presence of the following manifestations (PaO_2_ <60 mmHg on room air, ratio of PaO_2_ to inspired oxygen fraction <300, or oxygen saturation <90%) and required therapeutic intervention (oxygen therapy or mechanical ventilation) for more than 24 hours;

*^e^* Bleeding after surgery that required secondary surgical hemostasis;

*^f^* New onset atrial fibrillation or paroxysmal supraventricular tachycardia that necessitated medical treatment;

*^g^* Concentration of cardiac troponin I exceed the diagnostic criteria for myocardial infarction as well as new Q waves (lasts for 0.03 s) or continuous (4 days) abnormal ST-T segment;

*^h^* Dyspnea and elevated brain natriuretic peptide level necessitating diuresis and noninvasive mechanical ventilation;

*^i^* Requirement of continuous infusion of inotropic agents or vasoconstrictors to maintain MAP≥65mmHg after surgery;

*^j^* Persisted new focal neurologic deficit and confirmed by neurologic imaging;

*^k^* Lack of bowel movement, flatulence, and requirement of parenteral nutrition for more than 1 week after surgery;

*^l^* Extravasation of contrast agent in the body cavity or retroperitoneal space that required percutaneous drainage;

*^m^* Clinical manifestations combined with evidence from B ultrasound or CT scan;

*^n^* Elevation of serum transaminase level above 3 times the upper limit, excluded myocardial and skeletal muscle injury;

*^o^* Pus expressed from the incision, and bacteria cultured from the pus;

*^p^* Wound rupture that required secondary suturing;

*^q^* Confirmed by urinalysis and urine culture and necessitated antibiotic therapy;

*^r^* Defined as infection with acute change of SOFA score≥2, according to sepsis 3.0 diagnostic criteria;

*^s^* Symptoms of bleeding combined with prolonged prothrombin time and activated partial thromboplastin time, decreased fibrinogen and increased level of D-Dimer and fibrinogen degradation product;

*^t^* Decrease of hemoglobin level combined with positive gastrointestinal occult blood test results that required treatment;

*^u^* Pulmonary embolism: confirmed by CTPA;

*^v^* Deep venous thrombosis: confirmed by deep venous ultrasonography.
