# Supplementary material for: Preoperative hypoalbuminemia was associated with acute kidney injury in high-risk patients following non-cardiac surgery: a retrospective cohort study
Source: BMC Anesthesiol. 2019 Sep 2;19:171. doi: 10.1186/s12871-019-0842-3 (PMC6719349; doi:10.1186/s12871-019-0842-3)
Supplement: Supplementary file 3 — Figure S1. Preoperative serum albumin receiver operating characteristic curve for discriminating critically ill subjects with or without AKI. Demonstrates preoperative serum albumin receiver operating characteristic curve for discriminating patients with or without AKI. (DOCX 29 kb) [file 12871_2019_842_MOESM3_ESM.docx]

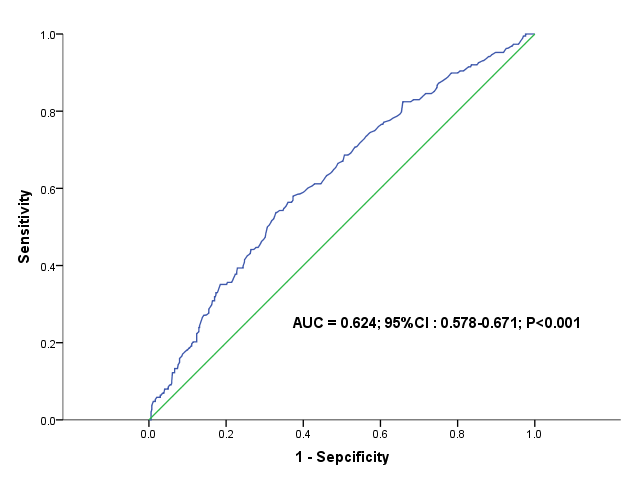


**Fig S1.** Preoperative serum albumin receiver operating characteristic curve for discriminating critically ill subjects with AKI (n = 188) from those without AKI (n = 541). The cutoff value of preoperative serum albumin for postoperative AKI was 37.5g/L determined by the Youden index with a sensitivity of 0.54, specificity of 0.67 and positive predictive value of 0.36. AUC, area under the curve; CI, confidence interval; AKI, acute kidney injury.
