# Supplementary material for: Preoperative hypoalbuminemia was associated with acute kidney injury in high-risk patients following non-cardiac surgery: a retrospective cohort study
Source: BMC Anesthesiol. 2019 Sep 2;19:171. doi: 10.1186/s12871-019-0842-3 (PMC6719349; doi:10.1186/s12871-019-0842-3)
Supplement: Supplementary file 4 — Table S3. Preoperative variables after propensity score matching. Description: Demonstrates preoperative variables between patients with or without hypoalbuminemia after propensity score matching. (DOCX 20 kb) [file 12871_2019_842_MOESM4_ESM.docx]

**Table S3. Preoperative variables after propensity score-matching**

|  | Total  (n=322) | Without postoperative AKI (n=244) | With postoperative AKI (n=78) | P value | Preoperative albumin ≥ 37.5g/L^a^ (n=161) | Preoperative albumin < 37.5g/L^a^ (n=161) | P value |
| --- | --- | --- | --- | --- | --- | --- | --- |
| Age (y) | 69±16 | 67±16 | 74±15 | 0.001 | 70±14 | 68±18 | 0.364 |
| Male sex | 178 (55.3%) | 132 (54.1%) | 46 (59.0%) | 0.451 | 83 (51.6%) | 95 (59.0%) | 0.179 |
| BMI (kg/m^2^) | 23.7±4.4 | 23.5±4.5 | 24.4±4.2 | 0.133 | 23.6±3.8 | 23.7±5.0 | 0.841 |
| Preoperative comorbidities |  | | | | | | |
| Diabetes mellitus | 86 (26.7%) | 63 (25.8%) | 23 (29.5%) | 0.524 | 43 (26.7%) | 43 (26.7%) | >0.999 |
| Hypertension | 176 (54.7%) | 127 (52.0%) | 49 (62.8%) | 0.096 | 92 (57.1%) | 84 (52.2%) | 0.370 |
| Coronary heart disease | 84 (26.1%) | 57 (23.4%) | 27 (34.6%) | 0.049 | 47 (29.2%) | 37 (23.0%) | 0.204 |
| Congestive heart failure | 13 (4.0%) | 7 (2.9%) | 6 (7.7%) | 0.091 | 8 (5.0%) | 5 (3.1%) | 0.396 |
| Cerebrovascular disease | 61 (18.9%) | 43 (17.6%) | 18 (23.1%) | 0.285 | 33 (20.5%) | 28 (17.4%) | 0.477 |
| Chronic kidney disease | 12 (3.7%) | 6 (2.5%) | 6 (7.7%) | 0.078 | 5 (3.1%) | 7 (4.3%) | 0.556 |
| Lung disease ^b^ | 32 (9.9%) | 25 (10.2%) | 7 (9.0%) | 0.744 | 16 (9.9%) | 16 (9.9%) | >0.999 |
| Liver disease ^c^ | 11 (3.4%) | 9 (3.7%) | 2 (2.6%) | >0.999 | 4 (2.5%) | 7 (4.3%) | 0.357 |
| Malignant neoplasm | 206 (64.0%) | 160 (65.6%) | 46 (59.0%) | 0.291 | 113 (70.2%) | 93 (57.8%) | 0.020 |
| Peripheral vascular disease | 17 (5.3%) | 12 (4.9%) | 5 (6.4%) | 0.570 | 11 (6.8%) | 6 (3.7%) | 0.213 |
| ASA classification |  | | | 0.004 |  | | 0.026 |
| I | 4 (1.2%) | 4 (1.6%) | 0 (0.0%) |  | 0 (0.0%) | 4 (2.5%) |  |
| II | 113 (35.1%) | 96 (39.3%) | 17 (21.8%) |  | 60 (37.3%) | 53 (32.9%) |  |
| III | 183 (56.8%) | 132 (54.1%) | 51 (65.4%) |  | 95 (59.0%) | 88 (54.7%) |  |
| IV | 22 (6.8%) | 12 (4.9%) | 10 (12.8%) |  | 6 (3.7%) | 16 (9.9%) |  |
| Preoperative Hb (g/L) ^d^ | 123±20 | 123±18 | 123±23 | 0.872 | 121±19 | 124±20 | 0.239 |
| Preoperative albumin (g/L) ^d^ | 37.2±5.5 | 37.8±5.4 | 35.3±5.5 | 0.001 | 41.5±2.7 | 32.8±4.1 | <0.001 |
| Preoperative albumin < 37.5g/L | 161 (50.0%) | 108 (44.3%) | 53 (67.9%) | <0.001 | __ | __ | __ |
| Baseline serum creatinine (umol/L) ^e^ | 79.8±27.9 | 78.0±24.7 | 85.4±35.9 | 0.041 | 82.5±28.3 | 77.2±27.4 | 0.088 |
| Preoperative BNP (pg/ml) ^f^ | 112 (56, 214) | 102 (52, 187) | 147 (77, 254) | 0.025 | 107 (52, 197) | 113 (56, 218) | 0.574 |
| Radiocontrast exposure ^g^ | 31 (9.6%) | 16 (6.6%) | 15 (19.2%) | 0.001 | 15 (9.3%) | 16 (9.9%) | 0.850 |
| On ACEI/ARB | 29 (9.0%) | 21 (8.6%) | 8 (10.3%) | 0.658 | 15 (9.3%) | 14 (8.7%) | 0.846 |
| Smoking habit ^h^ | 63 (19.6%) | 50 (20.5%) | 13 (16.7%) | 0.459 | 35 (21.7%) | 28 (17.4%) | 0.325 |

Data are presented as mean ± SD, median (interquartile range), or number of patients (percentage) and compared by independent samples t-test, Mann-Whitney U test or chi-squared test/Fisher’s exact test respectively.

ACEI, angiotensin converting enzyme inhibitor; ARB, angiotensin receptor blocker; ASA, American Society of Anesthesiologists; BMI, body mass index; BNP, B-type natriuretic peptide; cTNI, cardio-troponin; Hb, hemoglobin.

^a^ The cutoff value of preoperative albumin for postoperative AKI was determined by the Youden index of ROC curve [see Additional file 3: Fig S1].

^b^ Including chronic obstructive pulmonary disease (COPD), asthma and pulmonary fibrosis.

^c^ Including any kind of chronic hepatitis and liver cirrhosis.

^d^ Measured within 3 days before surgery.

^e^ Determined by the minimal value of serum creatinine measured within 3 months before admission and in hospital before surgery; if neither value was available, the modification of diet in renal disease formula was adopted to estimate the baseline serum creatinine according to the Kidney Disease Improving Global Outcomes guideline.

^f^ Measured in 177 patients before surgery.

^g^ Including patients who had radiocontrast exposure within 7 days before surgery.

^h^ Smoking for more than 10 cigarettes per day for more than 1 year, including current or past smokers.
