# Supplementary material for: Preoperative hypoalbuminemia was associated with acute kidney injury in high-risk patients following non-cardiac surgery: a retrospective cohort study
Source: BMC Anesthesiol. 2019 Sep 2;19:171. doi: 10.1186/s12871-019-0842-3 (PMC6719349; doi:10.1186/s12871-019-0842-3)
Supplement: Supplementary file 5 — Table S4. Intra- and postoperative variables after propensity score-matching. Demonstrates intra- and postoperative variables between patients with or without hypoalbuminemia after propensity score-matching. (DOCX 21 kb) [file 12871_2019_842_MOESM5_ESM.docx]

**Table S4. Intra- and postoperative variables after propensity score-matching**

|  | Total  (n=322) | Without postoperative AKI (n=244) | With postoperative AKI (n=78) | P value | Preoperative albumin ≥ 37.5g/L^a^ (n=161) | Preoperative albumin < 37.5g/L^a^ (n=161) | P value |
| --- | --- | --- | --- | --- | --- | --- | --- |
| Duration of anesthesia (min) | 257 (181, 333) | 255 (179, 336) | 259 (192, 330) | 0.618 | 243 (179, 318) | 273 (187, 367) | 0.041 |
| Duration of surgery (min) | 168 (106, 245) | 168 (105, 249) | 168 (106, 231) | 0.811 | 158 (105, 229) | 187 (106, 277) | 0.061 |
| Emergency surgery | 33 (10.2%) | 18 (7.4%) | 15 (19.2%) | 0.003 | 6 (3.7%) | 27 (16.8%) | <0.001 |
| Open surgery ^b^ | 133 (55.6%) | 95 (53.7%) | 38 (61.3%) | 0.299 | 53 (44.5%) | 80 (66.7%) | 0.001 |
| Type of surgery |  | | | | | | |
| General surgery ^c^ | 161 (50.0%) | 112 (45.9%) | 49 (62.8%) | 0.009 | 75 (46.6%) | 86 (53.4%) | 0.220 |
| Neurosurgery | 10 (3.1) | 8 (3.3%) | 2 (2.6%) | >0.999 | 4 (2.5%) | 6 (3.7%) | 0.521 |
| Thoracic surgery | 26 (8.1%) | 21 (8.6%) | 5 (6.4%) | 0.535 | 11 (6.8%) | 15 (9.3%) | 0.413 |
| Urologic surgery | 69 (21.4%) | 61 (25.0%) | 8 (10.3%) | 0.006 | 50 (31.1%) | 19 (11.8%) | <0.001 |
| Gynecological surgery | 12 (3.7%) | 8 (3.3%) | 4 (5.1%) | 0.494 | 3 (1.9%) | 9 (5.6%) | 0.078 |
| Orthopedic surgery | 23 (7.1%) | 16 (6.6%) | 7 (9.0%) | 0.471 | 9 (5.6%) | 14 (8.7%) | 0.279 |
| Vascular surgery | 1 (0.3%) | 1 (0.4%) | 0 (0.0%) | >0.999 | 0 (0.0%) | 1 (0.6%) | >0.999 |
| ENT surgery | 6 (1.9%) | 5 (2.0%) | 1 (1.3%) | >0.999 | 1 (0.6%) | 5 (3.1%) | 0.214 |
| Other ^d^ | 9 (2.8%) | 7 (2.9%) | 2 (2.6%) | >0.999 | 5 (3.1%) | 4 (2.5%) | >0.999 |
| Intraoperative maximal lactate (mmol/L) ^e^ | 1.0 (0.8, 1.5) | 1.0 (0.8, 1.5) | 1.0 (0.8, 1.5) | 0.582 | 1.0 (0.8, 1.4) | 1.1 (0.8, 1.6) | 0.191 |
| Intraoperative minimal Hb (g/L) ^e^ | 108 ± 25 | 106 ± 23 | 113 ± 28 | 0.036 | 105 ± 22 | 110 ± 27 | 0.073 |
| Intraoperative management |  | | | | | | |
| Use of vasopressors ^f^ | 79 (24.8%) | 55 (22.8%) | 24 (30.8%) | 0.158 | 37 (23.4%) | 42 (26.1%) | 0.581 |
| Volume of artificial colloid infusion (ml) | 500 (500, 1000) | 500 (500, 1000) | 500 (500, 1000) | 0.316 | 500 (500, 1000) | 500 (500, 1000) | 0.085 |
| Estimated blood loss (ml) | 100 (10, 300) | 100 (10, 300) | 100 (10, 200) | 0.953 | 100 (10, 200) | 100 (10, 300) | 0.382 |
| Positive fluid balance (ml) | 2173 ± 1619 | 2240 ± 1574 | 1979 ± 1741 | 0.229 | 1999 ± 1190 | 2333 ± 1922 | 0.072 |
| Postoperative variables before AKI ^g^ |  | | | | | | |
| Nephrotoxin exposure |  | | | | | | |
| Glycopeptides | 25 (7.8%) | 21 (8.6%) | 4 (5.1%) | 0.318 | 4 (2.5%) | 21 (13.0%) | <0.001 |
| Aminoglycoside | 0 (0.0%) | 0 (0.0%) | 0 (0.0%) | -- | 0 (0.0%) | 0 (0.0%) | -- |
| NSAIDs | 186 (57.8%) | 147 (60.2%) | 39 (50.0%) | 0.111 | 114 (70.8%) | 72 (44.7%) | <0.001 |
| Hemolysis | 0 (0.0%) | 0 (0.0%) | -- | 0 (0.0%) | 0 (0.0%) | 0 (0.0%) | -- |
| Rhabdomyolysis | 1 (0.3%) | 1 (0.4%) | 0 (0.0%) | >0.999 | 0 (0.0%) | 1 (0.6%) | >0.999 |
| Sepsis | 32 (9.9%) | 21 (8.6%) | 11 (14.1%) | 0.158 | 7 (4.3%) | 25 (15.5%) | 0.001 |
| Use of vasopressors ^f^ | 40 (12.4%) | 32 (13.1%) | 8 (10.3%) | 0.505 | 19 (11.8%) | 21 (13.0%) | 0.735 |
| Minimal Hb (g/L) | 99 ± 18 | 100 ± 19 | 98 ± 17 | 0.563 | 98 ± 19 | 100 ± 17 | 0.407 |
| Maximal BNP (pg/ml) | 275 (148, 491) | 280 (149, 497) | 264 (143, 444) | 0.719 | 257 (134, 491) | 287 (164, 474) | 0.265 |
| Maximal lactate (mmol/L) | 1.9 (1.3, 2.7) | 1.9 (1.3, 2.7) | 1.8 (1.3, 2.4) | 0.246 | 1.9 (1.4, 2.6) | 1.9 (1.3, 2.7) | 0.571 |
| Perioperative blood transfusion *^h^* | 83 (25.8%) | 62 (25.4%) | 21 (26.9%) | 0.790 | 38 (23.6%) | 45 (28.0%) | 0.372 |
| Non-renal SOFA within 24h ICU admission | 2 (1, 3) | 2 (1, 3) | 2 (2, 4) | 0.168 | 2 (1, 3) | 3 (2, 4) | 0.002 |
| Postoperative AKI | 78 (24.2%) | — | — | — | 25 (15.5%) | 53 (32.9%) | <0.001 |

Data are presented as mean ± SD, median (interquartile range), or number of patients (percentage) and compared by independent samples t-test, Mann-Whitney U test or chi-squared test/Fisher’s exact test respectively.

BNP, B-type natriuretic peptide; ENT, ear, nose and throat; Hb, hemoglobin; NSAIDs, non-steroidal anti-inflammatory drugs, SOFA, sequential organ failure assessment score.

^a^ The cutoff value of preoperative albumin for postoperative AKI was determined by the Youden index of the ROC curve [see Additional file 3: Fig S1].

^b^ Open or laparoscopic surgery referred to 239 patients.

^c^ Abdominal surgery, such as gastrointestinal, hepatobiliary and pancreatic surgery.

^d^ Thyroid or breast surgery.

^e^ Measured by arterial blood gas analysis.

^f^ Including use of phenylephrine, norepinephrine, epinephrine and dopamine.

^g^ Occurred before start of AKI.

*^h^* Perioperative blood products transfusion, including packed red blood cell, plasma and platelet.
