# Supplementary material for: Preoperative hypoalbuminemia was associated with acute kidney injury in high-risk patients following non-cardiac surgery: a retrospective cohort study
Source: BMC Anesthesiol. 2019 Sep 2;19:171. doi: 10.1186/s12871-019-0842-3 (PMC6719349; doi:10.1186/s12871-019-0842-3)
Supplement: Supplementary file 6 — Table S5. Independent risk factors for postoperative AKI after propensity score matching. Demonstrates the independent risk factors for postoperative AKI after propensity score matching. (DOCX 16 kb) [file 12871_2019_842_MOESM6_ESM.docx]

**Table S5. Independent risk factors for postoperative AKI after propensity score-matching**

|  | Univariate Logistic model ^a^ | | Multivariate Logistic model ^b^ | |
| --- | --- | --- | --- | --- |
|  | OR (95% CI) | P value | OR (95% CI) | P value |
| Age (y) | 1.031 (1.012-1.050) | 0.001 | 1.031 (1.009-1.053) | 0.005 |
| History of hypertension | 1.557 (0.922-2.627) | 0.098 | — | — |
| History of coronary heart disease | 1.737 (0.999-3.019) | 0.050 | — | — |
| History of congestive heart failure | 2.821 (0.919-8.663) | 0.070 | — | — |
| History of chronic kidney disease | 3.306 (1.034-10.564) | 0.044 | — | — |
| ASA classification | 2.236 (1.430-3.498) | <0.001 | 1.827 (1.085-3.075) | 0.023 |
| Radiocontrast exposure ^c^ | 3.393 (1.590-7.238) | 0.002 | 3.295 (1.408-7.712) | 0.039 |
| Baseline creatinine (umol/L) ^d^ | 1.009 (1.000-1.017) | 0.047 | 1.015 (1.004-1.026) | 0.009 |
| Preoperative albumin < 37.5g/L ^e^ | 2.670 (1.558-4.574) | <0.001 | 3.085 (1.649-5.771) | <0.001 |
| Emergency surgery | 2.989 (1.426-6.265) | 0.004 | — | — |
| General surgery ^f^ | 1.991 (1.180-3.362) | 0.010 | — | — |
| Intraoperative minimal Hb (g/L) ^g^ | 1.012 (1.001-1.023) | 0.038 | — | — |

ASA, American Society of Anesthesiologists; CI, confidence interval; Hb, hemoglobin; OR, odds ratio; SOFA, sequential organ failure assessment score.

^a^ Perioperative variables with P *<* 0.10 in the univariate analyses by independent samples t test, Mann-Whitney U test, chi-square test or Fisher's exact test were included.

^b^ Backward: LR.

^c^ Including patients who had radiocontrast exposure within 7 days before surgery.

^d^ Determined by the minimal value of serum creatinine measured within 3 months before admission and in hospital before surgery; if neither value was available, the modification of diet in renal disease formula was adopted to estimate the baseline serum creatinine according to the Kidney Disease Improving Global Outcomes guideline.

^e^ Measured by arterial blood gas analysis.

^f^ Abdominal surgery, such as gastrointestinal, hepatobiliary and pancreatic surgery.

^g^ Including use of phenylephrine, norepinephrine, epinephrine and dopamine.
